# Special Issue “New Insights into Current and Future Vaccines against SARS-CoV-2 Variants of Concern and Interest”

**DOI:** 10.3390/vaccines11030603

**Published:** 2023-03-06

**Authors:** Bingqian Qu, Xue Li

**Affiliations:** 1 Division of Veterinary Medicine, Paul Ehrlich Institute, 63225 Langen, Germany; 2 European Virus Bioinformatics Center, 07743 Jena, Germany; 3 Department of Internal Medicine III, University Hospital Heidelberg, 69120 Heidelberg, Germany

The coronavirus (COVID-19) pandemic has been a global threat for the past three years at the time of writing, leading to more than 675 million confirmed cases and 6.8 million deaths as of 28 February 2023. To date, treatments with several small-molecule antivirals and engineered antibodies have been authorized in the European Union. Despite these medications, prophylactic vaccination remains the most efficient method that builds immunity in individuals prior to infection. Although virus spread has decelerated worldwide, the battle is not over yet due to at least four reasons: (I) new variants as an uncertain threat; (II) the time cost of developing and licensing new vaccines; (III) the limited duration of neutralizing antibodies after vaccination and relatively weak mucosal immunity induced by the vaccine and (IV) vaccine hesitancy.

As of 31 May 2021, the World Health Organization (WHO) defined SARS-CoV-2 variants of concern (VOC), of interest (VOI) and high consequence or under monitoring. However, most approved vaccines, i.e., Comirnaty, Spikevax, Vaxzevria, Janssen COVID-19 vaccine, Nuvaxovid, inactivated COVID-19 vaccine (BIBP-CorV) and CoronaVac, were manufactured relying on the ancestral virus. Breakthrough infections with VOC have occurred in some people with vaccinations, although the severity of symptoms was alleviated, and the mortality rate was lower. Hence, more effective strategies for new vaccines should be developed in response to the constantly mutating viruses. To update, a bivalent mRNA vaccine composed of equal amounts of 15 μg of mRNA encoding wild-type and Omicron BA.4/BA.5 spike proteins was approved by the European Medicines Agency (EMA) in the fourth season of 2022.

In this Special Issue “New Insights into Current and Future Vaccines against SARS-CoV-2 Variants of Concern and Interest”, we have brought a broad range of new reports on COVID-19 vaccines covering development, safety and adverse effects, effectiveness, evaluation in special population and administration in developing countries. In addition, many challenges that remain unaddressed were highlighted.

*New possibility of vaccination development*. Viral vector vaccines have been developed through multiple platforms. The vectors include adenovirus (Vaxzevria), yellow fever virus, vaccinia virus and measles virus. However, the vector components could cause fever, headaches or muscle pain after intramuscular injection. Yoon et al. developed an oral vaccine candidate based on a *Salmonella* (*S.*) *Typhimurium* strain [1]. A plasmid vector expressing a modified spike antigen was transformed into *Salmonella*. The safety and stability of these recombinant *Salmonella* cells were evaluated in vitro by incubation with RAW264.7 murine macrophages and in vivo by oral administration in Balb/c mice. These *Salmonella* strains did not expand in the body when the mice were orally inoculated with a normal dose of bacteria. All the mice inoculated with the engineered *Salmonella* survived, demonstrating the safety of the bacterial vector. Furthermore, B and T lymphocytes were activated in response to the SipB60-spike fusion protein expressed and presented by the engineered *Salmonella*. One week after the last immunization, IgG2a subtype antibody levels were elevated and spike antigen-specific cytotoxic T cell responses were determined. However, it remains unclear whether the bacterial delivery induces a robust IgA antibody response in the gut mucosa, and how long the responses last before the bacteria are inactivated by the host immune system.

*Adverse effects in patients with immune disease*. Adverse reactions such as anaphylaxis, Guillain–Barré syndrome, myocarditis, pericarditis and thrombosis with thrombocytopenia syndrome have been reported with mRNA- and adenovirus-based COVID-19 vaccines in healthy vaccinees. A series of reports showed that autoimmune diseases, e.g., systemic lupus erythematosus may flare up after vaccination. In immune-mediated inflammatory disorder patients, the risk of disturbed immune responses is higher. Costanzo et al. reported a case of eosinophilic granulomatosis with polyangiitis (EGPA) experiencing a relapse after the first dose of the mRNA vaccine [2]. The patient with asthma was diagnosed with EGPA and treated subcutaneously with Mepolizumab. Three months later after recovering from SARS-CoV-2 infection, she received her first dose of the BNT162b2 mRNA vaccine, but then developed respiratory distress, myalgia and numbness of the limbs due to EGPA flare within ten days after vaccination.

In this case, it is unknown whether the spike proteins expressed from mRNA could interfere with angiotensin-converting enzyme 2 (ACE2), the SARS-CoV-2 receptor located in vascular endothelial cells. Such binding would alter the vascular microenvironment and mediate EGPA relapse. Furthermore, the robust immune reactions induced by mRNA vaccine may exaggerate the inflammatory response in the patient. Alternatively, the adjuvant may be the culprit of the exacerbation of inflammation. The activated immune responses may disrupt the immune balance controlled by Mepolizumab and push the body into a pro-inflammatory state. The exact cause of the adverse effect is still under investigation. Therefore, post-vaccination consequences in patients with autoimmune diseases should be meticulously monitored. How to activate immunity to COVID-19 without affecting immunosuppression in patients remains a challenge for vaccine development.

*Antibody dynamics after vaccination*. National vaccination programs in many countries showed how effective COVID-19 vaccines are in healthy people and how long the vaccine-mediated protective period is. With diagnostic tests, Skorupa, Szczepanek et al. evaluated the dynamics and durability of anti-SARS-CoV-2 spike IgG from the peripheral blood taken from 18,610 Polish healthcare workers every month until 12 months post-vaccination [3]. In the first 4 months post full vaccination, high levels of anti-SARS-CoV-2 IgG were detected with four different vaccines (BNT162b2, mRNA-1273, ChAdOx1 and Ad26.COV2.S).

Before vaccination, the antibody level in seropositive workers was higher than those in uninfected testers. The time span of anti-SARS-CoV-2 IgG levels lasted 12 months after two doses of vaccination and 1~3 months after booster dose [3]. Overall, 5~8 months post full vaccination, the IgG levels elicited by the tested vaccines were stable and comparable, except Ad26.COV2.S with significantly lower IgG levels. Post hoc assessment revealed that antibody levels for 3 months after vaccination were relatively high, then the antibody gradually eclipsed in the next 6 to 9 months. SARS-CoV-2 infection or booster vaccination afterward was able to increase levels of antibodies again. In addition, the third dose successfully induced antibodies in those people who did not respond to the first two doses. Participants with prior SARS-CoV-2 infection had significantly higher antibody levels than uninfected ones.

Whether current vaccines have sufficient efficacy over months for protection against SARS-CoV-2 VOC is still under investigation and discussion. Do we need to keep vaccinating people with updated vaccines at a regular period, such as in the case of influenza vaccine development? The decision on vaccination frequency undoubtedly requires more relevant reports and studies.

*Evaluation of maternal responses in pregnant women*. Due to the physiological changes in the immune and cardiopulmonary systems, pregnant women are associated with a higher risk of severe COVID-19 infection and more adverse outcomes. They shall receive special attention during the pandemic. At the beginning of the epidemic, pregnant women were excluded from vaccination clinical trials due to safety concerns because of a high risk of maternal and neonatal complications. Inadequate data on the efficacy and safety of vaccination in this special group was collected. Benefits and risks of a pregnant or breast-feeding woman and her embryo, fetus or baby after birth should be evaluated in COVID-19 vaccine development. Clinical data have reinforced confidence in the vaccine safety and efficacy for pregnant women. Since infants have an incompletely developed immune system, COVID-19 vaccination is not permitted in most countries. They need to receive maternal antibodies from their mothers and become protected. It is still an option for pregnant women to be vaccinated to establish protection for both the maternal body and the baby after birth.

Liu et al. reviewed the transplacental transfer of maternal anti-SARS-CoV-2 antibodies and analyzed the influencing factors of maternal antibody transfer according to the published work [4]. Upon SARS-CoV-2 infection, pregnant women had a high placental transfer rate, which allowed fetuses to acquire maternal antibodies. However, anti-SARS-CoV-2 IgG levels in neonates declined rapidly or disappeared within 6~11 weeks after birth. Maternal antibodies induced by COVID-19 vaccination can also be effectively transferred to fetuses. The IgG titers in newborns born to vaccinated mothers were significantly higher than in those born to infected mothers. Most infants of mothers who received COVID-19 vaccination compared with viral infection had longer-lasting antibodies after 6 months.

The dose of vaccination affects the efficiency of antibody production and the rate of maternal antibody transfer. Compared with those who had only one dose, a higher percentage of pregnant women with two-dose vaccination induced IgG antibodies. The IgG levels in pregnant women receiving a booster were significantly higher than those who received two doses. The maternal antibody transfer rate is related to the time interval between the last dose of vaccine and delivery. Follow-up studies can focus on the safe dose of vaccination for pregnant women. It remains unclear how robust these transferred antibodies protect infants and how durable this protection is.

*Vaccination program in Zimbabwe*. So far, more than 5 billion people worldwide have been fully vaccinated (65.4% of the population). However, the majority of these vaccinations were administrated in developed countries, whereas many sub-Saharan African countries remain behind, especially those countries not part of the COVID-19 Vaccines Global Access (COVAX) initiative. As an example, Zimbabwe is adjacent to South Africa where Beta and Omicron variants were first reported. Zimbabwe started a vaccination program in February 2021 aiming at vaccination for at least 60% of its population. However, the goal was not fulfilled as planned. Until today only 40.3% of the population has received the first dose and 29.7~31.3% (from different reports) is fully vaccinated.

Murewanhema et al. reviewed the landscape of COVID-19 vaccination in Zimbabwe and analyzed strengths, weaknesses, opportunities and threats of the program [5]. Pre-existing international connections among governments enabled Zimbabwe to access donated vaccines in the early months of the pandemic. Healthcare and customs staff were the first groups to receive vaccination. When more vaccines were available, the vaccination program further covered patients with chronic diseases, school staff and subsequently people over 18 years old. In addition, a Zimbabwe Expanded Program on Immunization (ZEPI) was established for alternative sources of vaccines. Through a series of collaborations with WHO and UNICEF, platforms have been established for training, surveillance and evaluation. These platforms in service enabled data collection and provided vaccination statistics as part of the COVID-19 global data. The execution of the programs in Zimbabwe inspired more collaborations between organizations including authorities, commercial companies, public health affiliates as well as international health organizations.

However, vaccine hesitancy remains a major challenge in the vaccination programs in Zimbabwe. To dispel the misconceptions and fiction about viruses and vaccines require more effort. Another obstacle is the lack of vaccines in Zimbabwe. People who are willing to be vaccinated sometimes cannot be vaccinated in time. This occurred frequently in poor urban and rural regions. Moreover, some people are willing to receive certain vaccines such as Comirnaty, Spikevax and Vaxzevria, but these vaccines are not yet available in Zimbabwe. There is still a long way to further a global vaccination program and it will require closer collaborations of people and organizations around the world.

*Concluding remarks*. This Special Issue has uncovered many of the pressing issues in the development and evaluation of the COVID-19 vaccines. However, these challenges are being addressed by rising enthusiasm in this field. At the beginning of 2023, there is renascent hope for the end of the pandemic, because the immune shield is being built when a high percentage of the population is vaccinated. We sincerely thank all contributing authors for their endeavors and hope our readers enjoy this Special Issue as much as we have enjoyed editing it.
